# Lightweight Compound Scaling Network for Nasopharyngeal Carcinoma Segmentation from MR Images

**DOI:** 10.3390/s22155875

**Published:** 2022-08-05

**Authors:** Yi Liu, Guanghui Han, Xiujian Liu

**Affiliations:** 1 School of Biomedical Engineering, Shenzhen Campus of Sun Yat-sen University, Shenzhen 518107, China; 2 Sun Yat-sen University, Guangzhou 510275, China; 3 School of Information Engineering, North China University of Water Resources and Electric Power, Zhengzhou 450046, China

**Keywords:** lightweight, nasopharyngeal carcinoma, deep learning, medical image segmentation

## Abstract

Nasopharyngeal carcinoma (NPC) is a category of tumours with a high incidence in head-and-neck. To treat nasopharyngeal cancer, doctors invariably need to perform focal segmentation. However, manual segmentation is time consuming and laborious for doctors and the existing automatic segmentation methods require large computing resources, which makes some small and medium-sized hospitals unaffordable. To enable small and medium-sized hospitals with limited computational resources to run the model smoothly and improve the accuracy of structure, we propose a new LW-UNet network. The network utilises lightweight modules to form the Compound Scaling Encoder and combines the benefits of UNet to make the model both lightweight and accurate. Our model achieves a high accuracy with a Dice coefficient value of 0.813 with 3.55 M parameters and 7.51 G of FLOPs within 0.1 s (testing time in GPU), which is the best result compared with four other state-of-the-art models.

## 1. Introduction

Among cancers of the head and neck [1], nasopharyngeal carcinoma is one of the most common types [2]. Nasopharyngeal carcinoma (NPC) is a highly invasive neoplasia that spreads early to regional lymph nodes. NPC is common in southern China, the Middle East, and North Africa. It has significant geographical variation and gender differences [3]. NPC has the highest incidence in Southeast Asia in up to 6.4/100,000 males and 2.4/100,000 females in these regions [4]. In 2012, the mortality of nasopharyngeal carcinoma (NPC) reached 58.6%, and in 2018 there were about 130,000 incidents in the world, including more than 73,000 people dying from nasopharyngeal carcinoma [5].

Nasopharyngeal carcinoma is mainly treated with radiotherapy. Currently, the delineation of the tumour area in radiotherapy is generally performed by manual segmentation. Doctors segment the MR images of nasopharyngeal carcinoma to match the target area and tumour extent as accurately as possible, so that radiotherapy can obtain better effects. However, manual segmentation costs a lot of time and energy for doctors [6], and the accuracy of manual segmentation is also affected by the experience of doctors. Although some automatic segmentation models have emerged to assist physicians in nasopharyngeal carcinoma segmentation, the existing models have a large number of parameters and consume many computing resources. Small and medium-sized hospitals cannot afford the large resource consumption and therefore still use manual segmentation for nasopharyngeal carcinoma treatment. To solve the above problems caused by manual segmentation, we proposed a lightweight automatic segmentation method for nasopharyngeal carcinoma.

With the advancement of convolutional neural networks, the research on medical image processing based on semantic segmentation has developed rapidly. Semantic segmentation is divided into two categories. Traditional image segmentation uses grayscale, color, texture, shape, and other features to divide the image into regions so that there are obvious differences and similarities between regions. Tatanun et al. [7] and Huang et al. [8] used the threshold method, region growth method, statistical theory, and other traditional image segmentation methods to segment nasopharyngeal carcinoma tumours. In addition, machine learning methods such as SVM [9,10,11] and SOM [12] are also used to segment nasopharyngeal carcinoma tumours. However, the traditional methods mentioned above usually require manual intervention processes such as feature extraction and dimension reduction; this has disadvantages such as poor model robustness and noise sensitivity.

The emergence of deep learning-based methods has led to significant changes in the approach to the field of computer vision [13,14,15,16,17,18,19,20,21]. Deep learning is used in a wide variety of tasks in computer vision and medicine [22,23,24,25,26], where neural networks consisting of an encoder-decoder framework have become one of the dominant models in deep learning. Ji et al. [27] reviewed a CNN-based encoder-decoder framework for a significant object detection neural network in recent years, showing its achievements and great potential in the field of salient object detection. Chen et al. [28] built PAD-Net by using an encoder-decoder framework, which has achieved great success in the field of stereoscopic image quality measurement (SIQM). In addition, the encoder-decoder framework has also achieved success in damage detection [29], scene independent evaluation [30], material capture [31], handwriting recognition [32], etc. The automatic segmentation method of nasopharyngeal carcinoma is developed based on the second method to achieve the effect of assisting doctors in treatment and diagnosis.

**Figure 1:**
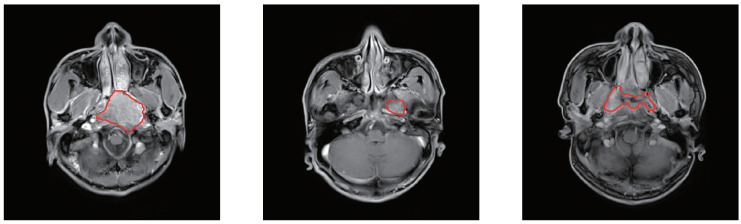
MRI slices of head and neck and tumour location of nasopharyngeal carcinoma. The area delineated by the red line is the result of the doctor’s manual segmentation.

However, the existing automatic segmentation methods for nasopharyngeal carcinoma face three challenges: First, nasopharyngeal carcinoma is adjacent to a few normal tissues and even infiltrates, such as mucosa, and its intensity range is almost the same as that of nasopharyngeal carcinoma [33]. In addition, the shape and size of lesions vary from patient to patient and the blurred boundaries of head and neck tumours on MRI are also a pain point (as shown in Figure 1) [8]. Second, unlike other medical image processing tasks that have a large number of similar images and have the same image quality, for the same NPC patient, the complicated shapes and remote location of the tumour may create more difficulties for segmentation. Third, the different hospitals provide different operating environment resources for the model. Some hospitals can only provide limited resources, so it is unavailable for them to run larger models.

Currently some classical convolutional neural networks are proposed and used for medical image segmentation of nasopharyngeal carcinoma, such as VGGNet [34], ResNet [35], FCN [36], SegNet [37]. In 2015, Olaf proposed a U-shaped network framework named UNet [38], it has greatly influenced the field of semantic segmentation, especially the field of medical image segmentation. Since UNet [38] can combine low-resolution information, which is conducive to the recognition of targets, and at the same time combines high-resolution information, it solves the disadvantages of blurred boundaries and complex gradients in medical images. After that, many UNet-based convolutional neural networks were proposed, such as Att-UNet [39], AttR2U-Net [40], Res-UNet [35] and DA-DSUNet [41] etc.

In addition to the UNet-based method, many other deep learning methods have been proposed and used for nasopharyngeal carcinoma medical image segmentation tasks. Li et al. [42] used the full convolution encoding-decoding neural network of 27 patients with NPC MR image segmentation and adopted it to realize the NPC automatic segment. Ma et al. [43] used a convolutional neural network to segment the focal regions of T1W mode MR images of 30 NPC patients, improved the segmentation results with a 3D image cutting algorithm, and finally achieved a relatively good result. However, the networks of the above two methods have the disadvantages of a simple structure, fewer experimental data, and the generality and accuracy of the model need to be improved. Moreover, the deployment of the network is too troublesome and not light enough for small and medium-sized hospitals.

In this paper, we propose a lightweight model named LW-UNet to solve the problems of the resource-constrained situation of small and medium-sized hospitals. We build the Compound Scaling Encoder inspired by EfficientNet [44]; this encoder improves the accuracy and reduces the parameters of nasopharyngeal carcinoma segmentation by compound scaling depth, width, and resolution. It enables small and medium-sized hospitals to run smoothly under resource constraints. The decoder is similar to UNet. Compared with other NPC segmentation models, our model has the following two advantages:

(1) Our model uses a fixed mixing coefficient to uniformly scale depth, width, and resolution to improve the accuracy and phase rate of the network;

(2) Our model uses lightweight modules to enable it to run under resource constraints.

The rest of the paper is structured as follows. In Section 2, we will introduce our proposed method. The dataset used in this research and the experimental details are described in Section 3. The results are discussed in Section 4.

## 2. Method

**Figure 2:**
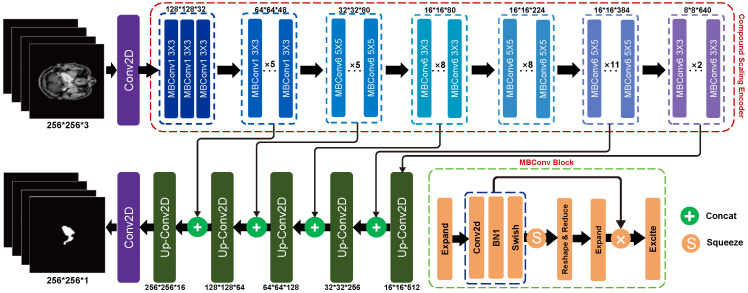
Network of LW-UNET: The Compound Scaling Encoder of LW-UNet consists of seven MBConv blocks of different sizes. Decoder is composed of a series of up-convolutions, and through skip connection and up-convolution can obtain the final segmentation map of nasopharyngeal carcinoma.

LW-UNet is a UNet-like model; the detailed network is shown in Figure 2. Our model consists of two parts: Compound Scaling Encoder and UNet-like decoder. Inspired by EfficientNet [44], we propose the Compound Scaling Encoder. It introduces scale coefficient to scale width, depth and resolution to reduce network parameters and FLOPs (See Section 2.1). We refer to UNet [38] and propose a decoder with a similar structure (See Section 2.2). Firstly our model utilizes the Compound Scaling Encoder to capture image information to obtain the feature maps. Then, the feature maps of the encoder are upsampled by up-convolution. The output of the 2, 3, 5, 6, 7 blocks of the encoder and up-convolution are concatenated by a skip connection. Finally, as the decoder continues to recover image resolution and detail, we obtain the segmentation maps with the same resolution as the original image.

### 2.1. Compound Scaling Encoder

The development of the convolutional neural network is usually based on a fixed resource budget [44]. If more resources are available, the size of the network can be expanded. A traditional CNN network typically scales the depth and width of the network and the size of the input images resolution to improve the accuracy, such as ResNet [35], GPipe [45]. However, the traditional method generally scales the width, depth and resolution randomly. It is difficult to adjust with limited resources.

In 2020, Tan et al. [44] proposed a new model scaling method, which utilizes a coefficient to uniformly scale the depth, width and resolution, rather than scale the single dimension of the network as in the traditional method. Tan introduced α, β, γ to measure the specific weights of depth, width, resolution, and θ as a scaling coefficient. He considers that if depth is doubled, the amount of calculation is doubled. However, if width or resolution are doubled, then the amount of calculation increases by four times. In other words, the amount of calculation is proportional to depth, width, and resolution. By referring to the method of Tan et al. [44], we utilize a compound scaling coefficient to uniformly scale the width, depth, and the resolution of the input image of the network. The compound scaling coefficients are utilized as in Equation (1):

(1) $$\begin{array}{c} D=k_{i}\times d\\ W=L_{i}\times \omega \\ R=r\times \gamma , \end{array}$$

where *D*, *W*, *R* represent the depth, width and resolution, $k_{i}$, $L_{i}$, *r* represent the *i*-th block kernel size, the *i*-th layer and the input size resolution respectively, and *d*, ω, γ represent the scaling coefficient. By changing the scaling coefficient, models with different depth, width, and resolution are obtained. We refer to the method in EfficientNet [44] and use NAS (neural architecture search) to search for the best parameters corresponding to depth, width, and resolution, respectively, and fix them to obtain the baseline model LW-UNet 0. On this basis, the fixed parameters are scaled uniformly to obtain the scaling coefficients shown in Table 1. The fixed mixing coefficients determine the number of MBConv modules per block within the composite scaling encoder (as shown in Figure 2) in the baseline model (LW-UNet 0). With the scaling coefficients, we vary the number of MBConv modules in each block to obtain LW-UNet 1–5.

In addition to the compound coefficient for depth, width, and resolution, the Compound Scaling Encoder utilizes the Mobile Inverted Bottleneck Convolution module to make the model more lightweight. MBConv [46] is a lightweight module designed specifically for resource-constrained environments. Unlike normal convolution modules, MBConv utilizes depthwise separable convolution. Standard convolution takes a $h_{i}\times w_{i}\times d_{i}$ input tensor $L_{i}$, and applies convolutional kernel $K\in R^{k}\times k\times d_{i}\times d_{i}$ to produce a $h_{i}\times w_{i}\times d_{j}$ output tensor $L_{j}$. So the standard convolutional layer has the computational cost of $h_{i}\cdot w_{i}\cdot d_{i}\cdot d_{j}\cdot k\cdot k$. Compared with normal convolution, depthwise separable convolutions have almost the same effect but only cost $h_{i}\cdot w_{i}\cdot d_{i}\left(k^{2}+d_{j}\right).$

MBConv also constructs the Bottleneck Residual Block by using a linear bottleneck and an inverted residual mechanism. First, in the layer with a small number of channels, the linear bottleneck replaces the ReLU activation layer with the linear transform, which reduces the large information spoilage caused by ReLU. Second, the inverted residuals extend the low-dimensional input to higher dimensions by pointwise, and then subsequently proceed to depthwise. It can enhance the transfer of information and gradients without increasing the computational cost excessively. Our model utilizes MBConv modules of different sizes to constitute the encoder. The encoder composed of MBConv can reduce the computational resources efficiently.

### 2.2. UNet-like Decoder

UNet is proposed to solve the problem of medical image segmentation [38]. It is frequently used in the baseline of the medical image segmentation task because of its excellent performance. UNet consists of two parts: encoder and decoder. In the conventional UNet, the decoder is nearly symmetric to the encoder. However, unlike UNet, our network is asymmetric and the encoder is deeper than the decoder. The detailed network is revealed in Figure 2. In our work, we utilize the feature map of the last block of the encoder to upsample, then we concatenate with the same spatial resolution feature map from encoder. Through up-convolution and concatenation with a corresponding feature map from the encoder, the decoder can combine the spatial information. Before upsampling again, the image has to go through 3 × 3 convolutional layers first. This process is repeated until the segmentation map is the same size as the original input image.

### 2.3. Evaluation Method

#### 2.3.1. Accuracy Evaluation

In the training process, we utilize binary cross-entropy loss to evaluate the performance of the model, as shown in Equation (2).

(2) $$\begin{array}{c} Loss=-\frac{1}{N}\left(y_{n}\times log\left(z_{n}\right)+\left(1-y_{n}\right)\times log\left(1-z_{n}\right)\right), \end{array}$$

where *N* represents the total number of pixels, $Z_{n}$ represents the probability of predicting the *n*-th sample as a positive example, and $Y_{n}$ represents the label of the *n*-th sample.

In the test process, we utilise the Dice coefficient as the metric to evaluate the accuracy of the model [47]. The Dice coefficient measures the consistency between the two regions, which is defined as Equation (3).

(3) $$\begin{array}{c} DSC=\frac{2TP}{2TP+FP+FN}, \end{array}$$

where TP, FP, FN represent the number of true positive, false positive and false negative pixels respectively.

We also use IoU, Jaccard similarity [48], precision, specificity and sensitivity to evaluate the segmentation effect. We will discuss our model performance in Section 5.

#### 2.3.2. Parameters Evaluation

Deep neural networks are widely used in machine vision tasks such as image classification and object detection with great success. However, due to the limitation of memory and computing power, deploying a neural network on embedded devices is still an enormous challenge. In order to evaluate the lightweight degree of the model, we use Pytorch-OpCounter to calculate the parameters and flops of the model compared with four other state-of-the-art models.

## 3. Experiments

### 3.1. Dataset Description

Our model uses the NPC dataset from a total of 92 patients diagnosed with nasopharyngeal carcinoma, which contain 735 MR images. All images are MR T1W+C images of head and neck scanned by the Siemens Aera MRI system (approximately 100 slices per patient). The resulting MR images are stored in Digital Imaging and Communications in Medicine (DICOM) file format. To utilize the raw data for 2D image segmentation, we cropped each image to the region of interest (ROI) to reduce unnecessary computing workload. The cropped image includes the nasopharyngeal carcinoma area and the rest of the head region. Given the low number of nasopharyngeal carcinoma data obtained, we applied data augmentation to the dataset. We used HorizontalFlip, ShiftScaleRotate and other methods to transform the original nasopharyngeal carcinoma data. Finally we obtained 3678 images. Eighty percent of the images were used for training, 10% for validation, and 10% for testing.

### 3.2. Data Preprocessing

Considering the difference in image quality of MR images captured by different imaging equipment, we normalized the data inputted to the model. The specific formula is as in Equation (4).

(4) $$\begin{array}{c} X_{output}=\frac{X_{i}-X_{min}}{X_{max}-X_{min}}, \end{array}$$

where $X_{output}$ is the normalised data, $X_{i}$ is the original data, $X_{max}$ and $X_{min}$ are the maximum and minimum values of the original data set respectively.

Several previous studies have shown that data normalisation can make the data distribution more uniform and make the model converge faster, resulting in an improved model performance [49,50]. Images after processing have a mean of 0 and a variance of 1, conforming to the standard normal distribution. It effectively solves the problem of different picture quality. After the data were normalised, we scaled our input images to 256 × 256 and randomly assigned them to the training, validation and test sets. Finally our model was trained with a hyperparameter value of 1 for batch_size.

### 3.3. Implementation Details

In the training process of LW-UNet, we used the Adam optimizer to implement the gradient descent method [51]. We also used LRfinder to find the best learning rate and used ReduceLROnPlateau provided by PyTorch to adjust the learning rate properly [52]. In order to solve the overfitting problem to some extent, we used dropout and other mechanisms. We trained the model with the shuffled nasopharyngeal carcinoma images with segmentation labels [53]. We promptly tested the model on the validation set at the end of each training epoch to adjust the hyper-parameter.

## 4. Result

### 4.1. Ablation Study

To verify the effectiveness of our model, we perform the ablation study by comparing four variants of our model and the baseline UNet. The results in Table 2 demonstrate the efficiency and accuracy of our model. It proves that our structure is effective for nasopharyngeal carcinoma segmentation. The test results of our model are as follows: The average Dice coefficient value obtained from the test is 0.813, the average Jaccard similarity is 0.695, the average IoU is 0.696, the average specificity is 0.998 , the average precision is 0.787 and the average sensitivity is 0.824. We select four models LW-UNet 0, LW-UNet 1, LW-UNet 2 and LW-UNet 3 for comparison with UNet [38]. The results show that with the increasing width, depth and resolution of the model, the accuracy of the model increases continuously. Compared with LW-UNet 4, the DSC, JC, and IoU values of LW-UNet 3 are 0.02, 0.04, and 0.02 lower than those of LW-UNet 4, respectively, and the SE values are 0.1 higher than those of LW-UNet 4. Compared with LW-UNet 5, the DSC, JC, IoU, and SE values of LW-UNet 3 are respectively higher by 0.09, 0.06, 0.08, and 0.04. The LW-UNet 3 we use is superior to the LW-UNet 0, 1, 2 and 5 in terms of accuracy. The values of LW-UNet 4 are essentially the same as LW-UNet 3 at DSC, and some of the metrics are lower than LW-UNet 3. From the point of view of lightweight and accuracy, we finally chose LW-UNet 3 as our segmentation model. Starting from LW-UNet 1, our model has exceeded UNet with a higher accuracy. Compared with UNet, the LW-UNet-3 model increases DSC by 5%, IoU, JC and PC by 12%, 9% and 10% respectively.

Additional to this, we add experiments with the model to the original dataset. We processed MR images obtained from 92 patients diagnosed with nasopharyngeal carcinoma in the same way as the dataset obtained by data augmentation. We conducted the same experiments on both datasets using LW-UNet 0, 1, 2 and 3. By comparing the results with the experiments under data augmentation, we demonstrated the effectiveness of the data obtained by data augmentation in the case of fewer nasopharyngeal carcinoma data. The detailed experimental results are shown in Table 3. Compared to the values of the model in the original dataset, the DSC increased by 0.064, 0.073, 0.078 and 0.078 for LW-UNet 0, 1, 2 and 3, respectively, and the JC values increased by 0.06, 0.081, 0.124 and 0.112. In addition, for IoU, the values of LW-UNet 0, 1, 2 and 3 increased by 16%, 21% , 22% and 21%. 21%, 22%, 21%, for SE, the values of LW-UNet 0, 1, 2 and 3 increased by 20%, 19%, 10% and 6%, respectively.

### 4.2. Comparison with State-of-the-Art Models

#### 4.2.1. Comparison of Accuracy

**Figure 3:**
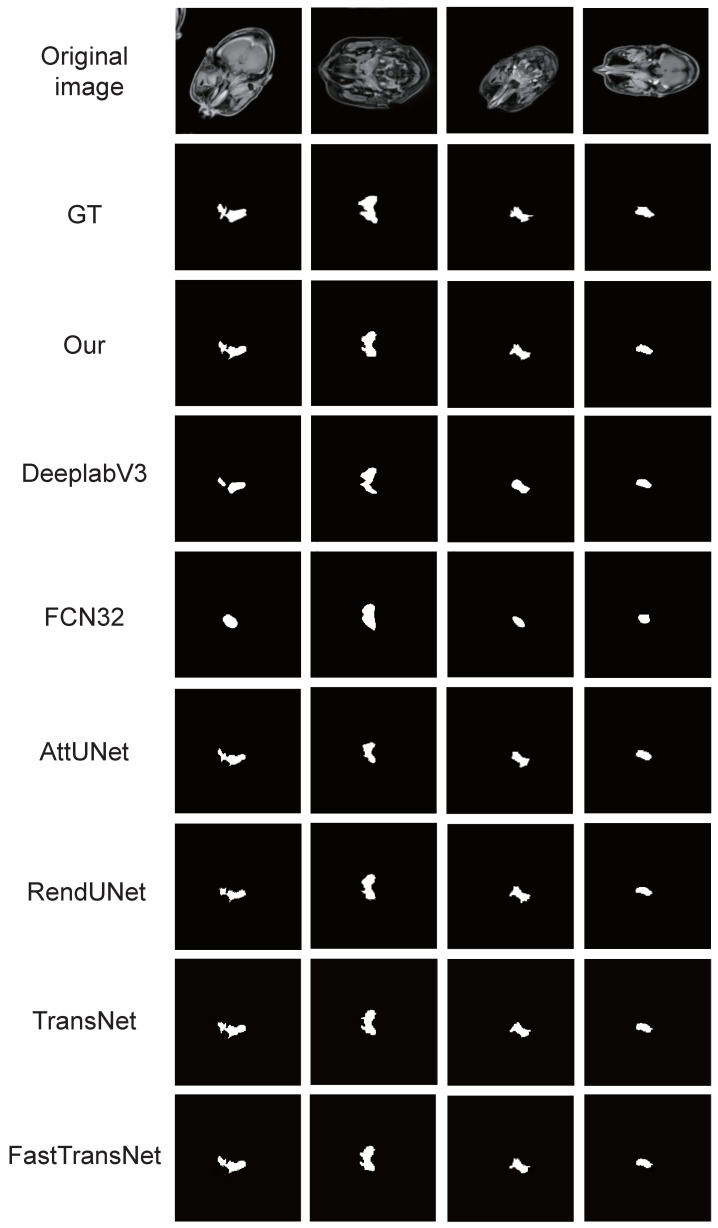
Examples of NPC segmentation results: We select four typical MRI images of nasopharyngeal carcinoma and present the segmentation results of our model and six models used for comparison.

**Figure 4:**
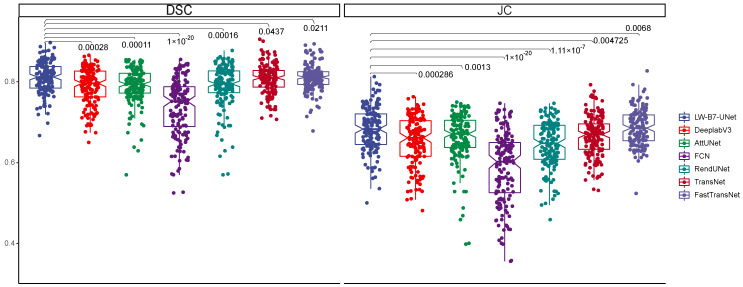
Box plots of the DSC and JC values obtained from the tests on the test set and its Kruskal–Wallis results. The results show that our model achieves the highest DSC and JC values in the test of nasopharyngeal carcinoma segmentation and is significantly different from other models.

We compare the accuracy of our model with four state-of-the-art models and carried out the Kruskal–Wallis test. Five of them are conventional medical image segmentation models (Att-UNet [39], FCN [36], DeeplabV3 [54], TransNet [55], FastTransNet [56]), and the other is a nasopharyngeal cancer image segmentation model (RendUNet [57]). Figure 3 shows the segmentation results for each model. Figure 4 shows the performance of each model on the DSC and JC metrics and Kruskal–Wallis test result. We achieved the highest DSC and JC value in the nasopharyngeal carcinoma segmentation test.

In DSC and JC metrics, our model outperforms the above four comparison models for nasopharyngeal carcinoma segmentation. For DeepLabV3, the average DSC value is 0.788 ± 0.045; we are 3.17% higher than it, and the average Jaccard similarity value is 0.654 ± 0.059; we are 6.26% higher than it. For Att-UNet, the average DSC value is 0.787 ± 0.047; we are 3.30% higher than it, the average Jaccard similarity value is 0.661 ± 0.061, we are 4.23% higher than it. For FCN, the average DSC value is 0.735 ± 0.072, we are 10.61% higher than it; the average Jaccard similarity value is 0.586 ± 0.087, we are 17.60% higher than it. For RendUNet, the average DSC value is 0.789 ± 0.054, we are 3.04% higher than it; the average Jaccard similarity value is 0.643 ± 0.058, 8.08% lower than ours. For TransNet, the average DSC value is 0.807 ± 0.036, ours 0.7% higher than it, the average Jaccard similarity value is 0.689 ± 0.051, ours 0.87% higher than ours. For FastTransNet, the average DSC value is 0.810 ± 0.033, ours 0.37% higher. The average Jaccard similarity value is 0.698 ± 0.045.

**Figure 5:**
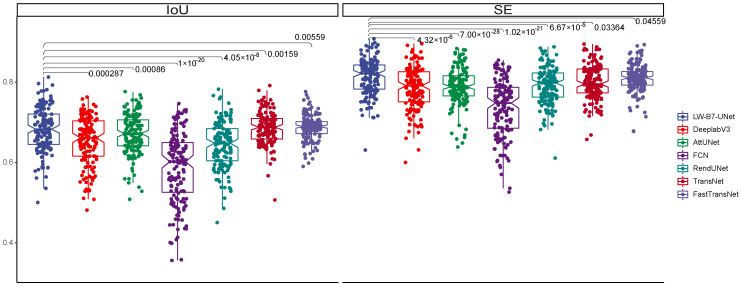
Box plots of the SE and IoU values obtained from the tests on the test set and its Kruskal–Wallis results. The results show that our model achieves the highest SE and IoU values in the test of nasopharyngeal carcinoma segmentation and is significantly different from other models.

We also compare other accuracy indicators of our model with four state-of-the-art models. Figure 5 shows the performance of each model on IoU and SE metrics. As we can see in Figure 5, we achieve the highest value in the nasopharyngeal carcinoma segmentation test.

As for other accuracy metrics, our model outperforms the above four comparison models for nasopharyngeal carcinoma segmentation. For DeepLabV3, the average IoU value is 0.653 ± 0.060, we are 6.58% higher than that, and the average sensitivity value is 0.787 ± 0.053, 4.70% lower than ours. For Att-UNet, the average IoU value is 0.671 ± 0.050, we are 2.68% higher; the average sensitivity value is 0.789 ± 0.046, we are 4.43% higher. For FCN, the average IoU value is 0.586 ± 0.088, we are 18.77% higher; the average sensitivity value is 0.735 ± 0.071, we are 12.10% higher. For RendUNet, the average IoU value is 0.643 ± 0.057, we are 8.24% higher; the average sensitivity value is 0.792 ± 0.045, we are 4.04% higher. For TransNet, the average IoU value is 0.691 ± 0.039, ours 0.71% higher; the average sensitivity value is 0.826 ± 0.074. For FastTransNet, the average IoU value is 0.692 ± 0.031, ours 0.57% higher than it, the average sensitivity value is 0.810 ± 0.033, ours 1.69% higher than it.

#### 4.2.2. Comparison of Parameters and FLOPs

As mentioned in Section 2.1 above, the LW-UNet model uses the MBConv module that enables the model to run under resource constraints. We compare the parameters and the FLOPs between our model and some advanced models, as shown in Table 4. For UNet [38], the parameters of our model are reduced by 872.67% and the FLOPs are reduced by 771.77%. For DeepLabV3 [54], the parameters of our model are reduced by 330.34% and the FLOPs are reduced by 114.91%. For Att-UNet [39], the parameters of our model are reduced by 882.53% and the FLOPs are reduced by 786.41%. For FCN [36], our model’s parameters are reduced by 314.64% and the FLOPs are reduced by 167.24%. For RendUNet [57], the parameters of our model are reduced by 1190.14% and the FLOPs are reduced by 533.55%. For TransNet, the parameters of our model are reduced by 2865.33% and the FLOPs are reduced by 228.09%. For FastTransNet, the parameters of our model are reduced by 740.84% and the FLOPs are reduced by 172.97%. Compared with the above advanced models, it can be seen that our proposed model achieves the best performance. Under the circumstance of limited hospital resources and environment, our model can still run efficiently and provide doctors with a more accurate delineation of target areas.

## 5. Discussion

Deep learning has achieved success on a variety of computer vision tasks [58,59,60,61,62,63]. LW-UNet is a lightweight automatic segmentation algorithm for nasopharyngeal carcinoma (NPC) based on deep learning. The lightweight network is applied in two main applications. First, it is applied to batch prediction tasks that require a high speed response [64], such as tumour segmentation tasks for nasopharyngeal cancer, lung cancer etc. This task requires a high segmentation speed of the model, and the LW-UNet has small parameters, consumes less computational power and can provide a low latency model to meet the needs of the hospital task. Secondly, the lightweight network is also applied to complete real-time low-latency segmentation tasks with limited computational storage resources or even on the mobile side [65]. Especially for small and medium-sized hospitals, it is able to complete real-time segmentation tasks at high speed with low equipment configuration, reducing the resource consumption of hospitals. LW-UNet has a low dependence on computational resources through a uniform scaling network of width, depth and resolution to improve the accessibility of the algorithm to small and medium-sized hospitals.

In previous research on medical imaging [66,67,68,69,70,71], deep learning technology is generally applied to simple organs such as the pancreas [72], liver [73] and lung [74]. However, the segmentation of nasopharyngeal carcinoma is more complex. Nasopharyngeal carcinoma tumour cells are generally located in the nasopharynx. The surrounding tissue structure is relatively complex, so the high segmentation accuracy is imperative. Because slightly inaccurate segmentation may cause damage to the patient’s brain.

In Figure 3, in the case of some simple nasopharyngeal carcinoma tumour, the results of the automatic segmentation of LW-UNet are the most similar to manual segmentation. However, LW-UNet is slightly limited in some complex cases because of unusual tumour shape and remote tumour location. In comparison with four other advanced models (including RendUNet [57], which specializes in nasopharyngeal carcinoma segmentation), our model achieved the best segmentation performance. In addition, our models are more effective with the lower parameters and FLOPs; this enables our model to operate in some small and medium-sized hospitals with limited resources. Considering the accuracy requirement of the segmentation, our model is temporarily unable to complete the segmentation task independently, so doctors need to check and modify it.

## 6. Conclusions

LW-UNet is an automatic segmentation model of nasopharyngeal carcinoma (NPC) proposed to solve the problems and difficulties of the problems of high computing resource requirements and low accuracy. By unifying the width, depth, and resolution of the scaling network, the model reduces the parameters and flops to improve the precision and efficiency of segmentation. Compared with other advanced models, our model achieve the best performance. It is expected to play a role in the future of nasopharyngeal carcinoma treatment. 

## Figures and Tables

**Table 1:** The scaling coefficient of LW-UNet 0–3.

Models	Depth Scaling Coefficient	Width Scaling Coefficient	Resolution Scaling Coefficient
LW-UNet 0	1.0	1.0	1.0
LW-UNet 1	1.4	1.2	1.3
LW-UNet 2	2.2	1.6	2.0
LW-UNet 3	3.1	2.0	2.7
LW-UNet 4	3.6	2.2	3.0
LW-UNet 5	4.1	2.4	3.4

**Table 2:** The ablation analysis validates the effectiveness of our model configuration.

Models	DSC	IoU	JC	SE	PC	SP
UNet	0.769 ± 0.063	0.618 ±0.058	0.632 ±0.076	0.858 ± 0.076	0.713 ± 0.089	0.996 ± 0.002
LW-UNet-0	0.696 ± 0.035	0.516 ± 0.043	0.542 ± 0.044	0.674 ± 0.035	0.623 ± 0.067	0.986 ± 0.001
LW-UNet-1	0.771 ± 0.041	0.621 ± 0.058	0.634 ± 0.058	0.801 ± 0.058	0.765 ± 0.059	0.997 ± 0.001
LW-UNet-2	0.796 ± 0.060	0.685 ±0.035	0.685 ±0.052	0.815 ± 0.048	0.767 ±0.053	0.998 ± 0.001
LW-UNet-3 (Our)	0.813 ± 0.039	0.696± 0.055	0.695 ± 0.055	0.824 ± 0.044	0.787± 0.043	0.998 ± 0.001
LW-UNet-4	0.815 ± 0.075	0.698 ± 0.043	0.699 ± 0.043	0.814 ± 0.011	0.787± 0.056	0.998 ± 0.001
LW-UNet-5	0.806 ± 0.054	0.688 ± 0.027	0.689 ± 0.076	0.820 ± 0.058	0.774± 0.084	0.998 ± 0.001

**Table 3:** The ablation analysis validates the effectiveness of our data augmentation.

Models	DSC	IoU	JC	SE	PC	SP
LW-UNet-0	0.632 ± 0.081	0.443 ± 0.028	0.482 ± 0.056	0.561 ± 0.048	0.545 ± 0.059	0.976 ± 0.001
LW-UNet-0 (with dataset augmentation)	0.696 ± 0.035	0.516 ± 0.043	0.542 ± 0.044	0.674 ± 0.035	0.623 ± 0.067	0.986 ± 0.001
LW-UNet-1	0.698 ± 0.071	0.513 ± 0.098	0.553 ± 0.078	0.668 ± 0.038	0.626 ± 0.079	0.984 ± 0.002
LW-UNet-1 (with dataset augmentation)	0.771 ± 0.041	0.621 ± 0.058	0.634 ± 0.058	0.801 ± 0.058	0.765 ± 0.059	0.997 ± 0.001
LW-UNet-2	0.718 ± 0.053	0.561 ± 0.076	0.561 ±0.055	0.736 ± 0.088	0.647 ±0.043	0.988 ± 0.001
LW-UNet-2 (with dataset augmentation)	0.796 ± 0.060	0.685 ±0.035	0.685 ±0.052	0.815 ± 0.048	0.767 ±0.053	0.998 ± 0.001
LW-UNet-3	0.735 ± 0.053	0.574 ± 0.068	0.583 ±0.096	0.775 ± 0.064	0.686 ± 0.085	0.995 ± 0.003
LW-UNet-3 (with dataset augmentation)	0.813 ± 0.039	0.696± 0.055	0.695 ± 0.055	0.824 ± 0.044	0.787± 0.043	0.998 ± 0.001

**Table 4:** Comparison Parameters and FLOPs with Other Models.

Category	Models	Parameters (M)	FLOPs (G)
Ablation Study Models	LW-UNet 5	7.36	9.01
	LW-UNet 4	5.32	8.38
	LW-UNet 3 (Our)	3.55	7.51
	LW-UNet 2	2.22	4.75
	LW-UNet 1	1.27	2.99
	LW-UNet 0	0.85	1.85
	UNet	34.53	65.47
State-of-the-art Models	DeepLabV3	15.31	16.14
	Att-UNet	34.88	66.57
	FCN32	14.72	20.07
	RendUNet	45.80	47.58
	FastTransNet	29.85	20.50
	TransNet	105.28	24.64

## Data Availability

The data presented in this study are available on request from the corresponding author. The data are not publicly available due to patient privacy.
